# Brain “fog,” inflammation and obesity: key aspects of neuropsychiatric disorders improved by luteolin

**DOI:** 10.3389/fnins.2015.00225

**Published:** 2015-07-03

**Authors:** Theoharis C. Theoharides, Julia M. Stewart, Erifili Hatziagelaki, Gerasimos Kolaitis

**Affiliations:** ^1^Laboratory of Molecular Immunopharmacology and Drug Discovery, Department of Integrative Physiology and Pathobiology, Tufts University School of MedicineBoston, MA, USA; ^2^Departments of Internal Medicine, Tufts University School of Medicine and Tufts Medical CenterBoston, MA, USA; ^3^Psychiatry, Tufts University School of Medicine and Tufts Medical CenterBoston, MA, USA; ^4^Sackler School of Graduate Biomedical Sciences, Tufts University School of MedicineBoston, MA, USA; ^5^Second Department of Internal Medicine, Attikon General Hospital, Athens Medical SchoolAthens, Greece; ^6^Department of Child Psychiatry, University of Athens Medical School, Aghia Sophia Children's HospitalAthens, Greece

**Keywords:** brain, cognition, cytokines, fog, histamine, inflammation, luteolin, mast cells

## Abstract

Brain “fog” is a constellation of symptoms that include reduced cognition, inability to concentrate and multitask, as well as loss of short and long term memory. Brain “fog” characterizes patients with autism spectrum disorders (ASDs), celiac disease, chronic fatigue syndrome, fibromyalgia, mastocytosis, and postural tachycardia syndrome (POTS), as well as “minimal cognitive impairment,” an early clinical presentation of Alzheimer's disease (AD), and other neuropsychiatric disorders. Brain “fog” may be due to inflammatory molecules, including adipocytokines and histamine released from mast cells (MCs) further stimulating microglia activation, and causing focal brain inflammation. Recent reviews have described the potential use of natural flavonoids for the treatment of neuropsychiatric and neurodegenerative diseases. The flavone luteolin has numerous useful actions that include: anti-oxidant, anti-inflammatory, microglia inhibition, neuroprotection, and memory increase. A liposomal luteolin formulation in olive fruit extract improved attention in children with ASDs and brain “fog” in mastocytosis patients. Methylated luteolin analogs with increased activity and better bioavailability could be developed into effective treatments for neuropsychiatric disorders and brain “fog.”

## Introduction

Brain “fog” is a constellation of symptoms that include reduced mental acuity and cognition, inability to concentrate and multitask, as well as loss of short and long-term memory. Brain “fog” characterizes patients with many neuroimmune diseases (Theoharides, 2013a) with celiac disease (Lebwohl and Ludvigsson, 2014; Lichtwark et al., 2014) chronic fatigue syndrome (Ocon, 2013), fibromyalgia and tachycardia postural syndrome (POTS) (Ross et al., 2013), as well as those with autism spectrum disorders (ASDs) and “minimal cognitive impairment,” which is now considered the early clinical presentation of Alzheimer's disease (AD) (Drzezga et al., 2011). Moreover, patients on chemotherapy often experience brain “fog” (Raffa, 2011).

Brain “fog” is particularly common in patients with systemic mastocytosis (SM) (Theoharides et al., 2015c) or disorders of mast cell (MC) activation (Valent et al., 2012; Petra et al., 2014). A recent survey of the symptoms experienced by patients with MC disorders reported that >90% of them experienced moderate to severe brain “fog” almost daily (Moura et al., 2012) and cognitive impairment was confirmed using a validated instrument (Moura et al., 2012). Patients with MC disorders also experience other related neurologic (Smith et al., 2011) and psychiatric (Moura et al., 2014) symptoms. It is interesting that children with mastocytosis were reported to have increased risk of developing ASDs compared to the general population (Theoharides, 2009). Children with ASDs are also characterized by brain “fog” (Rossignol and Frye, 2012) and focal brain inflammation (Theoharides et al., 2013) with MC activation being implicated in their pathogenesis (Theoharides et al., 2012a; Theoharides, 2013b).

Even though AD has typically been associated with brain senile plaques and neurofibrillary tangles that involve amyloid-β (Aβ) and tau proteins (Heneka et al., 2015), recent evidence indicates that oxidative stress/mitochondrial dysfunction (Zhu et al., 2012) and inflammation (Tan and Seshadri, 2010; Pizza et al., 2011; Heneka et al., 2015), are possibly involved in AD. In fact the immune system and inflammation are increasingly implicated in neuropsychiatric diseases (Kerr et al., 2005; Schmidt et al., 2007; Hamdani et al., 2013; Jones and Thomsen, 2013; Munkholm et al., 2013).

## Pathogenesis/focal inflammation

Inflammatory molecules, secreted in the brain could contribute to the pathogenesis of such diseases (Theoharides et al., 2004b) possibly including brain “fog.” Brain expression of pro-inflammatory genes was increased in the brains of deceased patients with neuropsychiatric diseases (Theoharides et al., 2011b).

It is still not clear what triggers brain inflammation. Mounting evidence suggests that stress (Theoharides et al., 2011b) and exposure to mold (Crago et al., 2003; Shoemaker and House, 2006; Reinhard et al., 2007; Shenassa et al., 2007; Empting, 2009), especially airborne mycotoxins (Rea et al., 2003; Gordon et al., 2004; Kilburn, 2009; Brewer et al., 2013), may be involved. It is interesting that mold can potentiate histamine release from MCs (Larsen et al., 1996).

**Figure 1:**
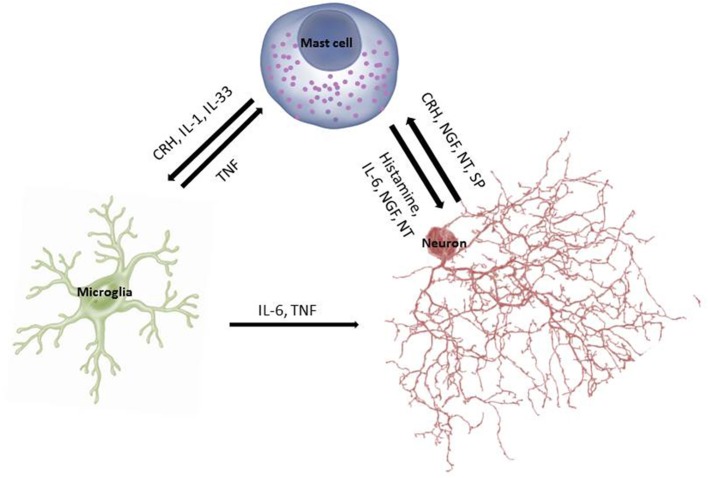
**Schematic representation of the cross-talk between mast cells, microglia, and neurons**.

In fact, cross-talk between MCs and microglia is being considered critical in the pathogenesis of neurodegenerative diseases (Skaper et al., 2012, 2013) (Figure 1). Microglia activation is a common finding in brains of children with ASDs (Pardo et al., 2005; Sandoval-Cruz et al., 2011; Gupta et al., 2014), as well as in other psychiatric diseases (Beumer et al., 2012). Activation of microglia directly or indirectly by corticotropin-releasing hormone (CRH) could contribute to the pathogenesis of mental disorders (Kritas et al., 2014b).

## Obesity

Obesity has been associated with neuropsychiatric disorders (Severance et al., 2012; Byrne et al., 2015). Adipocytokines are involved in neuroinflammation (Aguilar-Valles et al., 2015) and possibly in dementia (Arnoldussen et al., 2014; Kiliaan et al., 2014) including AD (Mathew et al., 2011; Khemka et al., 2014).

MCs have been implicated in obesity (Theoharides et al., 2011a), obesity-related asthma (Sismanopoulos et al., 2013) and in cardiovascular disease (CAD) (Alevizos et al., 2013; Chrostowska et al., 2013), which involves local inflammation (Libby et al., 2002; Matusik et al., 2012; Spinas et al., 2014). Both MCs (Kovanen et al., 1995; Laine et al., 1999) and histamine (Sakata et al., 1996) have been reported to be increased in atherosclerotic coronary plaques (Theoharides et al., 2011a). MC-derived histamine is a coronary constrictor. MC-derived IL-6 and TNF are independent risk factors for CAD (Libby et al., 2002) and can be released from MCs under stress (Huang et al., 2003), which can precipitate myocardial infarction (Alevizos et al., 2013). Obesity leads to endothelial dysfunction and chronic inflammation (Iantorno et al., 2014), also associated with the metabolic syndrome (Sun et al., 2015).

## Role of mast cells

MCs derive from bone marrow progenitors, mature in tissues depending on microenvironmental conditions and are critical for the development of allergic reactions, but also immunity (Galli et al., 2008b; Theoharides et al., 2010a; Sismanopoulos et al., 2012), neuroinflammation (Theoharides and Cochrane, 2004; Theoharides et al., 2010a; Skaper et al., 2012), and mitochondrial health (Theoharides et al., 2011b; Zhang et al., 2011). MCs can produce both pro- and anti-inflammatory mediators rendering capable to exert immuno-modulatory functions (Galli et al., 2008a; Kalesnikoff and Galli, 2008).

MCs are present in the brain where they regulate blood-brain barrier (BBB) permeability (Theoharides, 1990) and brain function (Nautiyal et al., 2008). MCs are located adjacent to CRH-positive neurons in the rat median eminence (Theoharides et al., 1995) and regulate the HPA axis (Theoharides et al., 2004a; Theoharides and Konstantinidou, 2007).

In addition to IgE and antigen (Blank and Rivera, 2004), MCs are activated by substance P (SP) (Zhang et al., 2011), neurotensin (NT) (Donelan et al., 2006), and nerve growth factor (NGF) (Kritas et al., 2014a). In fact, allergic MC stimulation leads to secretion of Hemokin 1, which acts in an autocrine manner through MC NK1 receptors to augment IgE-mediated allergic responses (Sumpter et al., 2015). MC stimulation by SP is augmented by IL-33 (Theoharides et al., 2010b), which has been considered an “alarmin” acting through MCs to alert the innate immune system (Moussion et al., 2008; Enoksson et al., 2011). IL-33 has been linked to autoimmune and inflammatory diseases (Theoharides et al., 2015c), especially brain inflammation (Chakraborty et al., 2010) and recently AD pathogenesis (Xiong et al., 2014). Antigen can also act synergistically with toll-like receptors (TLR-2 and TLR-4) to produce MC cytokines (Qiao et al., 2006) and regulate responses to pathogens (Abraham and St John, 2010; Theoharides, 2015).

Once activated, MCs secrete numerous vasoactive, neurosensitizing and pro-inflammatory mediators (Theoharides et al., 2015a). These include preformed histamine, serotonin, kinins, proteases and tumor necrosis factor (TNF), as well as newly synthesized, leukotrienes, prostaglandins, chemokines (CCXL8, CCL2), cytokines (IL-4, IL-6, IL-1, TNF) and vascular endothelial growth factor (VEGF), which increase BBB permeability (Theoharides et al., 2008). MCs store pre-formed TNF in secretory granules from which it is released rapidly (Zhang et al., 2012b) and stimulates activated T cells (Nakae et al., 2006; Kempuraj et al., 2008).

MCs can release some mediators, such as IL-6, selectively without degranulation (Theoharides et al., 2007). In addition, CRH can stimulate selective release of VEGF (Cao et al., 2005) and IL-1 can stimulate selective release of IL-6 (Kandere-Grzybowska et al., 2003), which could affect brain function (Theoharides et al., 2004a) and activate the HPA axis (Kalogeromitros et al., 2007). MC-derived IL-6 along with TGFβ stimulate development of Th-17 cells (Nakae et al., 2007) and MCs, themselves secrete IL-17 (Nakae et al., 2007), which is involved in autoimmunity. Levels of IL-6 were increased in the cerebrospinal fluid (CSF) (Li et al., 2009b) and plasma (Yang et al., 2015) of patients with ASDs. MCs can therefore participate in neuroinflammation (Theoharides and Cochrane, 2004; Zhang et al., 2012a; Dong et al., 2014), especially autism (Theoharides et al., 2012a, 2015b; Theoharides, 2013b).

Maternal administration of the viral substitute poly (I:C) produced autism-like behavior in mice that was dependent on IL-6 (Hsiao et al., 2012) and was absent in IL-6 knock-out mice (Smith et al., 2007). We had shown that acute immobilization stress significantly increased serum IL-6 and this was absent in MC deficient mice (Huang et al., 2003). It was recently reported that plasma IL-6 was significantly increased after social stress, especially in mice that developed a phenotype susceptible to stress, while IL-6^−∕−^ mice were resilient to social stress (Hodes et al., 2014).

MCs can secrete the content of individual granules (Theoharides and Douglas, 1978), and biogenic amines such as serotonin selectively without degranulation (Theoharides et al., 1982). MCs can communicate with neurons by transgranulation (Wilhelm et al., 2005). It was recently shown that MCs can undergo “polarized” exocytosis of proteolytic enzymes is what has been termed “antibody-dependent degranulation synapse” (Joulia et al., 2015). MCs can also secrete phospholipid nanovesicles (exosomes) (Skokos et al., 2002) that could cary a number of biologically active molecules (Shefler et al., 2011), in a manner guided by surface antigens (Bryniarski et al., 2013). Such exosomes could participate in neuropsychiatric diseases (Tsilioni et al., 2014; Kawikova and Askenase, 2015). In fact, individual MCs have been shown to exhibit “circadian clock” reactivity (Molyva et al., 2014; Nakao et al., 2015).

## Histamine

MCs are located perivascularly in close proximity to brain neurons especially in the leptomeninges (Rozniecki et al., 1999a) and hypothalamus (Pang et al., 1996) where they contain most of the brain histamine (Alstadhaug, 2014). Increasing evidence indicates that brain histamine is involved in the pathogenesis of neuropsychiatric diseases (Haas et al., 2008; Shan et al., 2015) and the disruption of the BBB (Banuelos-Cabrera et al., 2014), through MC activation (Esposito et al., 2001, 2002; McKittrick et al., 2015). Histamine may be important for alertness and motivation (Zlomuzica et al., 2008; Torrealba et al., 2012), as well as cognition, learning and memory (Kamei and Tasaka, 1993; Alvarez et al., 2001; Rizk et al., 2004; da Silveira et al., 2013). For instancee, there was enhanced spatial learning and memory in histamine 3 (H3) receptor mice^−∕−^ (Rizk et al., 2004). Moreover, antagonism of the autoinhibitory H3 receptor improved memory retention (Orsetti et al., 2001). In fact, H3 antagonists are being considered for the treatment of cognitive disorders and AD (Brioni et al., 2011).

It appears that some histamine is necessary for alertness, learning and motivation, but too much histamine shuts the system down, in MCs and histaminergic neurons, by activating H3 autoinhibitory receptors leading to brain “fog” (Table 1).

**Table 1:** **Effect of histamine on brain function**.

**Histamine**	**Source**	**Mechanism**	**Cognition-learning-attention, motivation**	**Brain fog, Anxiety**
Low	Increased diamine oxidase activity	Activation of H3 autoinhibitory receptors shuts down histamine synthesis and release	++	N/A
Normal			+++	N/A
High	Mast cell secretion, histamine containing foods, gut bacterial histamine production	Excessive use of H1 receptor antagonists	+	+++

Brain histamine can be increased by triggers of brain MCs, by histamine-containing foods (Bodmer et al., 1999; Maintz and Novak, 2007; Schwelberger, 2010; Prester, 2011), histamine produced by bacteria (Landete et al., 2008), or overuse of H1 receptor antagonists that would shift histamine binding from H1 to H3 receptors leading to autoinhibition of histamine synthesis and release (Table 1). In fact, we had shown that in rats at least only brain MCs express functional H3 receptors (Rozniecki et al., 1999b), as evidenced by the fact that an H3 receptor agonist inhibited while at H3 receptor antagonist augmented histamine and serotonin release only from brain, but not peritoneal MCs.

## Beneficial effect of luteolin

Recent reviews have discussed the potential use of flavonoids for the treatment of neuropsychiatric (Jager and Saaby, 2011; Grosso et al., 2013) and neurodegenerative (Jones et al., 2012; Solanki et al., 2015) diseases including AD (Sheikh et al., 2012; Baptista et al., 2014; Mecocci et al., 2014; Vauzour, 2014).

**Figure 2:**
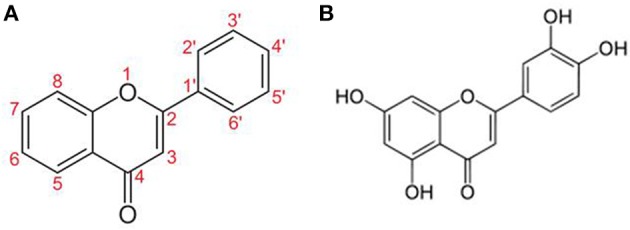
**structures of (A) Flavone and (B) Luteolin**.

Flavonoids (Figure 2) are naturally occurring compounds mostly found in green plants and seeds (Middleton et al., 2000). Unfortunately, our modern life diet contains progressively fewer flavonoids and under these conditions, the average person cannot consume enough to make a positive impact on health. Moreover, less than 10% of orally ingested flavonoids are absorbed (Passamonti et al., 2009; Thilakarathna and Rupasinghe, 2013) and are extensively metabolized to inactive ingredients in the liver (Chen et al., 2014).

Luteolin (5,7-3′5′-tetrahydroxyflavone) has potent antioxidant, anti-inflammatory (Middleton et al., 2000) and MC inhibitory activities (Kimata et al., 2000; Kempuraj et al., 2005; Asadi et al., 2010) and also inhibits auto-immune T cell activation (Verbeek et al., 2004; Kempuraj et al., 2008) (Table 2). Luteolin also inhibits microglial IL-6 release (Jang et al., 2008), microglial activation and proliferation (Chen et al., 2008; Dirscherl et al., 2010; Kao et al., 2011), as well as microglia-induced neuron apoptosis (Zhu et al., 2011).

**Table 2:** **Properties of the luteolin formulation**.

**LUTEOLIN**
Reduces oxidative stress
Inhibits inflammation
Inhibits mast cell activation
Inhibits microglia activation
Reduces LDL oxidation
Inhibits neurotoxicity
Memory
Mimics BDNF
Inhibits acetylcholinesterase
Prevents autism-like behavior in mice
Improves attention and sociability in children with ASDs
**OLIVE FRUIT EXTRACT**
Olive oil increases spatial memory
Hydroxytyrosol increases short-term memory
Oleocanthal inhibits amyloid-induced neurotoxicity

A methylated luteolin analog (6-Methoxyluteolin) was shown to inhibit IgE-stimulated histamine release from human basophilic KU812F (Shim et al., 2012). Moreover, we recently showed that tetramethoxyluteolin is more potent inhibitor of human cultured MCs than luteolin (Weng et al., 2014).

Luteolin is protective against methylmercury-induced mitochondrial damage (Franco et al., 2010), as well as mercury and mitochondrial DNA-triggering of MCs (Asadi et al., 2010).

Luteolin improved spatial memory in a scopolamine-induced model (Yoo et al., 2013) and in amyloid β-peptide-induced toxicity (Liu et al., 2009) in rats. Luteolin was also shown to induce the synthesis and secretion of neurotrophic factors in cultured rat astrocytes (Xu et al., 2013). The related flavonoid 7,8-dihydroxyflavone mimicked the activity of brain-derived neurotrophic factor (BDNF) (Jang et al., 2010b). Moreover, the related flavonoids 4′-methoxyflavone and 3′,4′-dimethoxyflavone were shown to be neuroprotective (Fatokun et al., 2013). Luteolin also protected again cognitive dysfunction induced by chronic cerebral hypoperfusion is rats (Hagedorn et al., 2010; Fu et al., 2014) and high fat-diet-induced cognitive dysfunction in mice (Liu et al., 2014). Furthermore, luteolin (Liu et al., 2009; Jang et al., 2010a; Yoo et al., 2013) increased memory and inhibited autism-like behavior in a mouse model of autism (Parker-Athill et al., 2009). The luteolin structurally related flavonol quercetin protected against amyloid β-induced neurotoxicity (Liu et al., 2013; Regitz et al., 2014) and improved cognition in a mouse model of AD (Wang et al., 2014). In fact, quercetin-o-glucuronide reduced the generation of β-amyloid in primary cultured neurons (Ho et al., 2013).

A luteolin containing formulation significantly improved attention and behavior in children with autism (Theoharides et al., 2012b; Taliou et al., 2013). This dietary supplement contains luteolin (100 mg per softgel capsule, >98% pure) formulated in olive fruit extract (<0.001 oleic acid acidity and water content), which increases oral absorption.

Olive fruit extract contains hydroxytyrosol, which has been reported to protect against brain hypoxia (Gonzalez-Correa et al., 2008) and oleocanthal, which inhibits fibrilization of tau proteins (Li et al., 2009a) and reduces aggregation of Aβ oligomers (Pitt et al., 2009) implicated in AD. Moreover, olive oil (Mohagheghi et al., 2010) and olive leaf extract (Mohagheghi et al., 2011) reduced BBB permeability. Data from animal studies indicate that use of olive oil (Tsai et al., 2007; Farr et al., 2012; Martinez-Lapiscina et al., 2013) increased memory.

Flavonoids have been proposed as possible therapeutic agents for CAD (Kempuraj et al., 2005; Perez-Vizcaino and Duarte, 2010; Yap et al., 2010). A meta analysis of epidemiological studies showed an inverse relationship between flavonol/flavone intake and CAD (Perez-Vizcaino and Duarte, 2010). A review of publications from European and US population cohorts reported that consumption of flavonoids was strongly associated with lower CAD mortality (Peterson et al., 2012). A double-blind, placebo-controlled, randomized clinical study using the polyhenolic compound Pycnogenol showed improved endothelial function in patients with CAD (Enseleit et al., 2012) and a study of 2-week consumption of a polyphenolic drink lowered urinary biomarkers of CAD (Mullen et al., 2011).

Luteolin suppressed adipocyte activation of macrophages, inhibited endothelial inflammation (Ando et al., 2009; Deqiu et al., 2011), increased insulin sensitivity of the endothelium (Deqiu et al., 2011), and prevented niacin-induced flush (Kalogeromitros et al., 2008; Papaliodis et al., 2008). Luteolin also protected low density lipoprotein from oxidation (Brown and Rice-Evans, 1998) and improved experimentally diet-induced obesity and insulin resistance (Xu et al., 2014), as well as protected against high fat-diet induced cognitive deficits (Liu et al., 2014) in mice.

## Mechanism of flavonoid action

Luteolin inhibits multiple signaling steps including PI3K, NFκB, PKCθ, STAT3, and intracellular calcium ions (Kempuraj et al., 2005; Lopez-Lazaro, 2009). Flavonoids also inhibit MC degranulation by interacting with distinct vesicle-dependent SNARE complexes (Yang et al., 2013). It was recently reported that certain flavonoids inhibited cytokine expression in mouse bone marrow-derived mast cell by interfering with IL-33 signaling (Funakoshi-Tago et al., 2015).

Flavonoids can also inhibit acetylcholinesterase (Tsai et al., 2007; Boudouda et al., 2015), which will increase acetylcholine and improve memory (Table 1). It is of interest that luteolin further inhibits release of the excitatory neurotransmitter glutamate (Lin et al., 2011), while it activates receptors for the inhibitory neurotransmitter γ-amino butyric acid (GABA) independent of GABA, suggesting it may also have a calming effect (Hanrahan et al., 2011). In fact, benzodiazepines that act by activating GABA receptors were shown to bind to MCs (Miller et al., 1988).

## Conclusion

Presently, 1 in 20 individuals over the age of 65 has dementia, while just the European population over 65 will rise from 17.4% in 2010 to 24% in 2030 or about 200 million people (United Nations Department of Economic and Social Affairs Population Division, 2015). The cost of caring for AD patients in the US is estimated to be $220 billion per year (Alzheimers Association, 2015). These numbers do not include brain “fog” present in the others disorders discussed. For instance, the cost of ASDs to the US economy is estimated at $ 180 billion per year. It is therefore obvious that any effective treatment will make a significant difference both to the health of the patients and to the economy. However, in spite of intensive research, clinical trials targeting *Aβ* have failed (Corbett et al., 2012) necessitating new therapeutic targets and there are no effective treatments for the other neuropsychiatric disorders discussed.

Flavonoids are generally considered safe (Kawanishi et al., 2005; Harwood et al., 2007; Seelinger et al., 2008; Corcoran et al., 2012; Theoharides et al., 2014). Unfortunately, some of the cheaper sources of flavonoids found in dietary supplements are from peanut shells and fava beans and may lead to anaphylactic reactions or hemolytic anemia to allergic and G_6_PD-deficient individuals, respectively. Flavonoids are extensively metabolized (Chen et al., 2014) primarily through glucoronidation, methylation, and sulphation (Hollman et al., 1995; Hollman and Katan, 1997). Therefore, flavonoids must be used with caution when administered with other natural polyphenolic molecules (e.g., curcumin, resveratrol) or drugs metabolized by the liver as they may affect the blood levels of themselves or of other drugs (Theoharides and Asadi, 2012). Tetramethoxyluteolin is already methylated and less likely to affect liver metabolism, is more stable (Walle, 2007), and has better bioavailability (Wei et al., 2014). Intranasal tetramethoxyluteolin preparations would offer the additional advantage of delivering the flavonoid directly to the brain through the cribriform plexus as was shown for some other compounds (Zhuang et al., 2011).

## Disclosures

TT is on the Scientific Advisory Board of the Mastocytosis Society (http://www.tmsforacure.org/) and on the Board of Directors of two nonprofit foundations (http://www.braingate.org; www.autismfreebrain.org). JS is the TMS regional patient support leader for Michigan. TT is the recipient of US Patent No. 8,268,365 for the treatment of brain inflammation, US Patent No. 7,906,153 for the treatment of multiple sclerosis, and US Patent No. 13/009.282 for the diagnosis and treatment of ASDs.

### Conflict of interest statement

The authors declare that the research was conducted in the absence of any commercial or financial relationships that could be construed as a potential conflict of interest.
